# α7 Nicotinic Acetylcholine Receptors May Improve Schwann Cell Regenerating Potential via Metabotropic Signaling Pathways

**DOI:** 10.3390/cells12111494

**Published:** 2023-05-28

**Authors:** Elisabetta Botticelli, Claudia Guerriero, Sergio Fucile, Maria Egle De Stefano, Carlo Matera, Clelia Dallanoce, Marco De Amici, Ada Maria Tata

**Affiliations:** 1 Department of Biology and Biotechnologies “Charles Darwin”, Sapienza University of Rome, Piazzale Aldo Moro, 5, 00185 Rome, Italy; botticellielisabetta9@gmail.com (E.B.); claudia.guerriero@uniroma1.it (C.G.); egle.destefano@uniroma1.it (M.E.D.S.); 2 IRCCS Neuromed, 86077 Pozzilli, Italy; sergio.fucile@uniroma1.it; 3 Department of Physiology and Pharmacology “V. Erspamer”, Sapienza University of Rome, 00185 Rome, Italy; 4 Research Centre of Neurobiology “Daniel Bovet”, Sapienza University of Rome, 00185 Rome, Italy; 5 Department of Pharmaceutical Sciences, University of Milan, 20133 Milan, Italy; carlo.matera@unimi.it (C.M.); clelia.dallanoce@unimi.it (C.D.);

**Keywords:** α7 nicotinic receptors, Schwann cells, metabotropic signals, mTORC1, cell migration

## Abstract

Background: Schwann cells (SCs) are glial cells involved in peripheral axon myelination. SCs also play a strategic role after peripheral nerve injury, regulating local inflammation and axon regeneration. Our previous studies demonstrated the presence of cholinergic receptors in SCs. In particular, the α7 nicotinic acetylcholine receptors (nAChRs) are expressed in SCs after peripheral axotomy, suggesting their involvement in the regulation of SC-regenerating properties. To clarify the role that α7 nAChRs may play after peripheral axon damage, in this study we investigated the signal transduction pathways triggered by receptor activation and the effects produced by their activation. Methods: Both ionotropic and metabotropic cholinergic signaling were analyzed by calcium imaging and Western blot analysis, respectively, following α7 nAChR activation. In addition, the expression of c-Jun and α7 nAChRs was evaluated by immunocytochemistry and Western blot analysis. Finally, the cell migration was studied by a wound healing assay. Results: Activation of α7 nAChRs, activated by the selective partial agonist ICH3, did not induce calcium mobilization but positively modulated the PI3K/AKT/mTORC1 axis. Activation of the mTORC1 complex was also supported by the up-regulated expression of its specific p-p70 S6K^Thr389^ target. Moreover, up-regulation of p-AMPK^Thr172^, a negative regulator of myelination, was also observed concomitantly to an increased nuclear accumulation of the transcription factor c-Jun. Cell migration and morphology analyses proved that α7 nAChR activation also promotes SC migration. Conclusions: Our data demonstrate that α7 nAChRs, expressed by SCs only after peripheral axon damage and/or in an inflammatory microenvironment, contribute to improve the SCs regenerating properties. Indeed, α7 nAChR stimulation leads to an upregulation of c-Jun expression and promotes Schwann cell migration by non-canonical pathways involving the mTORC1 activity.

## 1. Introduction

Myelinating glial cells, namely oligodendrocytes in the central nervous system (CNS) and Schwann cells (SCs) in the peripheral nervous system (PNS), are glial cell subpopulations playing a crucial role both during development and in the maintenance of the physiological functions of the mature nervous system. These cells perform multiple functions as they support neuronal survival and axon growth and overall contribute to myelin sheath formation. 

SCs develop from the neural crest, a transient multipotent cell population that is located at the dorsal side of the neural tube at the end of neuralization, able to generate several cell types. SCs are generated through a process that consists of two embryonic stages: the first one is gliogenesis, during which neural crest cells are committed in Schwann cell precursors (SCPs), followed by the acquisition of immature SC phenotypes. At the end of the embryonic stage, these cells can differentiate, leading to the formation of myelinating and non-myelinating SCs (so-called Remarck cells) [1,2]. SCs are characterized by remarkable plasticity and play a crucial role during the process of degeneration and regeneration of peripheral nerves. Like neurons, myelinating and non-myelinating SCs undergo gene expression changes during Wallerian degeneration, aimed at supporting the regenerating axons and controlling local inflammation. To acquire these new functions, SCs undergo a de-differentiation process during which they assume a particular phenotype called ”Repair Schwann Cells”. When the axonal regeneration is completed, they re-differentiate and form new myelin around the regenerated axons [1,3,4]. The relevance of SCs in regenerative processes has led to investigation of the cellular and molecular signals involved in the response to axonal damage, in view of developing therapeutic strategies able to improve and accelerate peripheral nerve regeneration. In recent years, several neurotransmitters have emerged as putative factors involved in SC development and differentiation. Among these, a crucial role is played by acetylcholine (ACh). Both rat and human SCs express muscarinic and nicotinic receptors able to modulate different responses to ACh stimuli [5,6,7,8,9]. Recently, we demonstrated that treatment with a selective M2 muscarinic acetylcholine receptor (mAChR) agonist may contribute to the inhibition of SC proliferation and migration and favor SC differentiation through the negative modulation of the PI3K/AKT/mTORC1 axis [9]. Furthermore, in previous studies we showed the lack of α7 nicotinic acetylcholine receptor (α7 nAChR) expression in SCs of rat sciatic nerve immediately after dissection, expression that was instead significantly enhanced after 24 h in both cultured sciatic nerve segments and in cultured SCs maintained in the presence of the pro-inflammatory neuropeptide bradykinin (BK). In addition, the selective activation of α7 nAChRs by the partial agonist ICH3 ((*R*)-(-)-3-methoxy-1-oxa-2,7-diaza-7,10-ethanospiro [4,5] dec-2-ene sesquifumarate) [10] reduced interleukin-6 production and increased metalloproteinase activity, promoting a microenvironment favorable to peripheral nerve regeneration [11].

α7 nAChRs are homopentameric ion channels largely expressed in the CNS. In the last fifteen years, in addition to their role as classical cholinergic receptors active at the nervous system synapses, they have been studied as modulators of the so-called cholinergic anti-inflammatory pathway in both immune as well as the nervous systems [12,13]. Although these ionotropic receptors mainly function as ion channels permeable to positive ions such as Na^+^, K^+^ and Ca^2+^, accumulating evidence suggests that α7 nAChRs may also modulate metabotropic pathways, particularly in non-neuronal cells [11,14]. On the other hand, it is known that the PI3K/AKT/mTORC1 pathway modulates the activity of SCs since different levels of mTORC1 can regulate crucial processes in SCs in different ways. In fact, low levels of mTORC1 promote a myelinating phenotype through the expression of the transcription factor Krox-20. Conversely, high but transitory levels of mTORC1, following nerve damage, are critical for the de-differentiation of SCs, through the expression of the transcription factor c-Jun [15,16]. Moreover, other kinases, such as AMPKα and PKCα, were found to be relevant for the acquisition of the ‘repair’ phenotype by SCs. AMPKα has been described as a negative regulator of myelination [17], while PKCα regulates the proliferation and migration of SCs following axonal damage [18]. Considering the morphological and functional properties of the SCs during axon damage and the peculiar expression of α7 nAChRs in SCs, notably after peripheral nerve injury [11], we investigated the ability of this receptor subtype to modulate the signaling pathways required for the acquisition and activity of the “Repair Schwann Cells” phenotype. To this end, we analyzed the effects of the ligand ICH3, a partial α7 nAChR agonist discovered and studied by our research group [19,20], which was previously characterized in ex vivo and in vitro rat SCs [11].

## 2. Materials and Methods

### 2.1. Statements for Animal Use

Procedures involving animals were performed in accordance with the guidelines of the Council of the European Communities Directive (86/609/EEC of 24 November 1986) and the Italian National Law DL/116/92. All methods were in accordance with the guidelines of Protocol No. 7FF2C.6.EXT.96 approved by the Ministry of Health (AMT, Aut. No. 1184/2016-PR 16/12/2016). All animals were housed in a temperature-controlled room (22 ± 1 °C) with a 12-h light/dark cycle and free access to food and water.

### 2.2. Cell Cultures

Primary Schwann cells were isolated from sciatic nerves of 2-day-old Wistar pups according to the protocol described in [7]. Briefly, sciatic nerves were harvested in Dulbecco’s Modified Eagle’s Medium (DMEM, Sigma-Aldrich, St. Louis, MO, USA) with the addition of Hepes 25 mM. Subsequently, sciatic nerves were treated with trypsin/collagenase (type I, Sigma-Aldrich, St. Louis, MO, USA) and mechanically isolated cells were seeded in T25 flasks with fresh DMEM containing 10% fetal bovine serum (FBS, Immunological Sciences, Milan, Italy). The removal of the fibroblasts was performed by treating the cells with 1 mM cytosine arabinoside (AraC, Sigma-Aldrich, St. Louis, MO, USA) for 48 h and then with anti-Thy 1.1 (1:1000, Serotec, Bio-Rad group, Hercules, CA, USA) and rabbit complement (1:2 *v*/*v*) (Cedarlane, Burlington, ON, Canada). Amplification of SCs was performed in DMEM, 10% FBS, 5 μM forskolin (Fsk; Sigma-Aldrich, St. Louis, MO, USA) and bovine pituitary extract (1:150, Sigma-Aldrich, St. Louis, MO, USA), keeping the cultures incubated in 10% CO_2_ at 37 °C and maintained at sub-confluent levels on 75 cm^2^ poly-lysine-coated flasks (Sigma-Aldrich, St. Louis, MO, USA). For the experiments, the cells were maintained in DMEM without sodium pyruvate, 10% FBS supplemented with 1% streptomycin, 50 IU/mL penicillin (Sigma-Aldrich, St. Louis, MO, USA), 1% glutamine (Sigma-Aldrich, St. Louis, MO, USA), 2 μM forskolin and 10 ng/mL Neuregulin-1 (Immunological Sciences, Milan, Italy).

### 2.3. Pharmacological Treatment

(*R*)-(-)-3-Methoxy-1-oxa-2,7-diaza-7,10-ethanospiro [4.5]dec-2-ene sesquifumarate (ICH3) was synthesized according to a published procedure [10], and was used at a final concentration of 10 μM. To mimic the inflammatory environment in vitro, SCs were pre-treated 24 h before the treatment with ICH3 with the pro-inflammatory peptide bradykinin (BK, Sigma-Aldrich, St. Louis, MO, USA), at a final concentration of 10 μM [11]. After 24 h, the medium with BK was removed, fresh medium with ICH3 was added, and the treatment at different time points was performed as required by the experimental plan.

### 2.4. [Ca^2+^]_i_ Measurements in Cultured SCs

Intracellular free Ca^2+^ concentration ([Ca^2+^]_i_) was measured using a microscopy system driven by Axon Imaging Workbench software (Molecular Devices, San Jose, CA, USA). Cells were incubated with the cell membrane permeant Fura-2 AM (4 μM, Molecular Probes, Life Technologies, Waltham, MA, USA ) for 45 min. Variation of [Ca^2+^]_i_ was expressed as time-resolved ratio, R, between fluorescence images obtained at 340 nm and 380 nm excitation wavelengths. Agonists were applied in the bath during recording, without further washing.

### 2.5. Total RNA Extraction and RT-PCR Analysis

Total RNA was extracted using TRI Reagent^®^ (Sigma-Aldrich, St. Louis, MO, USA), according to the manufacturer’s instructions. RNA was quantified using a NanoDrop^TM^ 2000 spectrophotometer (Thermo Fisher Scientific, Waltham, MA, USA). For each sample, 1 μg of total RNA was reverse transcribed using 5X All-In-One RT MasterMix with AccuRT Genomic DNA Removal Kit (Applied Biological Materials Inc., Richmond, CA, USA), according to the manufacturer’s instructions. For each sample, primers and GoTaq^®^ Green Master Mix (Promega Italia, Milan, Italy) were added to 100 ng of cDNA. The expression of the transcripts was evaluated by semi-quantitative RT-PCR analysis, using the following primers:

*α7*: forward 5′-AACCATGCGCCGTAGGACA-3′:

reverse 5′-CTCAGCCACAAGCAGCAGCATGAA-3′;

*gapdh*: forward 5′-TGGCATTGTGGAAGGGCTCATGAC-3′;

reverse 5′-ATGCCAGTGAGCTTCCCGTTCAGC-3′

### 2.6. Protein Extraction and Western Blot Analysis

Cells were lysed with lysis buffer (Tris-EDTA 10 mM, 0.5% NP40, NaCl 150 mM) containing a protease inhibitor cocktail (Sigma-Aldrich, St. Louis, MO, USA). Lysates were incubated for 20 min on ice, sonicated for 15 s, and then centrifuged for 10 min at 14,000 rpm at 4 °C. Protein concentration was determined using the BCA Protein Assay Kit (Thermo Fisher Scientific, Waltham, MA, USA). Samples containing sample buffer with 5% β-mercaptoethanol were heated for 5 min at 95 °C, loaded onto an 8% SDS-polyacrylamide gel (SDS-PAGE) and run at 100 V using running buffer (0.25 M Tris, 2.4 M Glycine, 0.035 M SDS). SDS-PAGE gels were transferred onto polyvinylidene difluoride (PVDF) sheets (Merck Millipore, Darmstadt, Germany) at 80 V in transfer buffer (20 mM Tris; 150 mM glycine, 5% [*v*/*v*] methanol) for 60 min at 4 °C. The membranes were blocked for 60 min with 5% non-fat milk powder (Sigma-Aldrich, St. Louis, MO, USA) in PBS containing 0.1% Tween-20 and then incubated at 4 °C overnight with the antibodies previously diluted in the blocking solution. The primary antibodies used were mouse anti-alpha7 antibody (Bioss, Woburn, MA, USA), rabbit anti-alpha3, -beta2 and -beta4 antibodies (courtesy from dr. C. Gotti to MEDS); anti PI3 Kinase p85 antibody (dilution 1:800, Cell Signaling Technology, Danvers, MA, USA), anti Phospho-AMPKα (Thr172) antibody (dilution 1:800, Cell Signaling Technology, Danvers, MA, USA), anti-AMPK𝛼1 (dilution 1:1000, Immunological Science, Milan, Italy), anti Phospho-AKT antibody (Thr308) (dilution 1:800, Cell Signaling Technology, Danvers, MA, USA), anti Phospho-AKT antibody (Ser473) (dilution 1:800, Cell Signaling Technology, Danvers, MA, USA), anti AKT (pan) (dilution 1:800, Cell Signaling Technology, Danvers, MA, USA), anti Phospho-p-70 S6 Kinase (T389) (dilution 1:600, Immunological Science, Rome, Italy), anti p-70 s6 Kinase (dilution 1:1000, Immunological Science, Rome, Italy), anti PKCα (dilution 1:2000, Immunological Science, Rome, Italy), c-Jun (dilution 1:1000, Cell Signaling Technology, Danvers, MA, USA), anti-FUS (dilution 1:1000, Santa Cruz Biotechnology, Dallas, TX, USA). β-actin (dilution 1:2000, Immunological Sciences, Milan, Italy) was used as a reference protein. After overnight incubation at 4 °C, the membranes were washed in PBS + 0.1% Tween-20 buffer and then incubated for 1 h at room temperature (RT) with the horseradish peroxidase-conjugated (HRP) secondary antibodies: rabbit anti-rabbit HRP (dilution 1:10000, Promega, Milan, Italy) or mouse anti-rat HRP (dilution 1:10,000, Immunological Sciences, Milan, Italy). Membranes were exposed to ECL chemiluminescence reagent (Immunological Sciences, Mi, Italy) for signal detection. The intensity of the bands was assessed by exposure to Chemidoc (Molecular Imager ChemiDoc XRS + System with Image Lab Software, Bio-Rad, Hercules, CA, USA). Densitometric analyses were performed using ImageJ imaging software (NIH, Bethesda, MD, USA).

### 2.7. Wound Healing Assay

The wound healing assay was used to evaluate cell migration. Cells were plated on 35 mm^2^ ∅ dishes and pre-treated with 10 μM BK for 24 h. Then cells were treated with 10 μM ICH3 for 48 h. When required, α-Bungarotoxin (αBTX, Tocris Bioscience, Bristol, UK) was supplied 2 h before ICH3. After 24 h of ICH3 treatment, the scratch was made with the p200 tip. The cells were photographed immediately after the scratch (T0) and after 6 h (T6) by an Axioskop 2 microscope (Zeiss, Oberkochen, Germany). The space between the two fronts at T0 and after 6 h was then measured using ImageJ software (NIH, Bethesda, MD, USA). The two values were subtracted (T0–T6), obtaining the covered space by the cells in the experimental time chosen.

### 2.8. Phalloidin Staining

SCs were plated on coverslips arranged in 24-well plates at the density of 2 × 10^4^ cells. Cells were pre-treated with 10 μM BK for 24 h. Then cells were treated with 10 μM ICH3 for 48 h. In the experiments with αBTX, this antagonist was supplied 2 h before ICH3. At the end of treatments, cells were washed 3 times with PBS and fixed with 4% paraformaldehyde in PBS for 20 min at RT. After 3 washes in PBS, cells were incubated with Phalloidin conjugated with Alexa Fluor™ 594 (Immunological Sciences, Milan, Italy) for 20 min to reveal actin filaments. After 3 washes in PBS, cells were incubated with Hoechst 33342 (1:1000 in PBS, Thermo Fisher Scientific, Waltham, MA, USA) for 10 min at RT, for the nuclei counterstaining. At the end, coverslips were fixed on microscope slides with a PBS-glycerol (3:1; *v*/*v*) solution. The images were acquired using an Axioskop 2 microscope (Zeiss, Oberkochen, Germany).

### 2.9. Immunocytochemistry

Schwann cells were placed on poly-lysine-coated coverslips placed in 24-well plates at a density of 2 × 10^4^ cells. Cells were pre-treated for 24 h with 10 μM BK followed by 48 h with the partial agonist ICH3 (10 μM). The cells were washed with PBS and fixed with 4% paraformaldehyde in PBS for 20 min at RT. After three washes in PBS, the cells were pre-incubated in a PBS solution containing 0.1% Triton X-100, 1% bovine serum albumin and 10% normal goat serum for 60 min at RT. The cells were then incubated with primary antibody c-Jun (dilution 1:400, Cell Signaling Technology, Danvers, MA, USA) at 4 °C overnight. Then the cells were washed with PBS and incubated with Alexa Fluor 488 Conjugated anti-rabbit secondary antibody (dilution 1:1000, Immunological Sciences, Milan, Italy) for 2 h at RT. Finally, nuclei were stained by using anti-fade mounting medium with 4′,6-diamidino-2-phenylindole (DAPI, Immunological Sciences, Milan, Italy). Images were acquired with a Zeiss Apotome fluorescence microscope, using a 63× objective through the Axion Vision programme (Carl Zeiss Inc., Oberkochen, Germany).

### 2.10. Nucleus–Cytoplasm Extraction

Schwann cells were plated on poly-lysine-coated 100 mm^2^ Ø dishes at a density of 1 × 10^6^ cells. The next day, cells were pre-treated with 10 μ BK M for 24 h. Then cells were treated with 10 μ ICH3 M for 24 h. The experimental condition with 100 ng/mL lipopolysaccharides (LPS, Sigma-Aldrich, St. Louis, MO, USA) was used as positive control. At the end of treatment, cells were scraped and they were collected after centrifugation. After washing in PBS, the cells were suspended in buffer A (10 mM Hepes, 10 mM KCl, 0.1 mM EDTA, 0.1 mM EGTA, 1 mM DTT, 0.5 mM PMSF, 1× Protease Inhibitor) and incubated on ice for 15 min. After, 10% NP40 was added in Buffer A and the samples incubated on ice for 5 min. The cells were centrifuged for at 1200× *g* for 5 min at 4 °C, and the supernatant (cytoplasmic extract) was stored in a new 1.5 ml tube. The pellet (nuclei) was diluted in buffer B (20 mM Hepes, 0.4 M KCl, 1 mM EDTA, 1 mM EGTA, 1 mM DTT, 1 mM PMSF, 1× Protease Inhibitor) with the addition of 5 M NaCl, vortexed vigorously and centrifuged at 15,000× *g* for 10 min at 4 °C. The supernatant (nuclear extract) was stored in a new 1.5 mL tube. Nuclear and cytoplasmic extracts were then analyzed by Western blot analysis. The cytoskeletal protein β-actin was used as a cytoplasmic marker, while Fused in Sarcoma (FUS), a nuclear RNA-binding protein, was used as a nuclear marker.

### 2.11. Statistical Analysis

Data analyses were performed with GraphPad Prism 9.1.0 (GraphPad Software Inc., La Jolla, CA, USA). Data are presented as the average ± SEM. Student’s *t*-test or one-way ANOVA analyses were used to evaluate statistical significance within the different samples. A value of *p* < 0.05 was considered statistically significant; *p* < 0.05 (*); *p* < 0.01 (**); *p* < 0.001 (***).

## 3. Results

### 3.1. Analysis of α7 nAChR Expression

**Figure 1:**
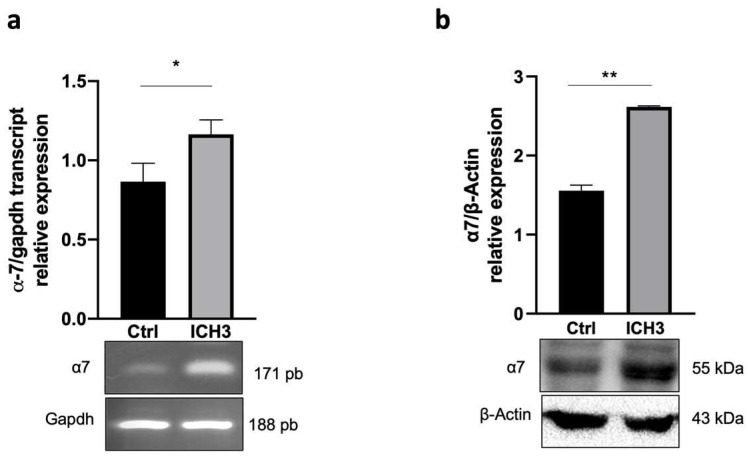
Expression levels of α7nAChRs in SCs. (**a**) RT-PCR analysis of the α7 nAChR transcript expression level in SCs after 48 h of 10 μM ICH3 treatment. SCs were pre-treated with BK for 24 h before ICH3 treatment. GAPDH was used as the housekeeping gene. The graph shows the densitometric analysis of the bands obtained from three independent experiments, normalized with the housekeeping gene. Student’s *t*-test was used to compare the samples (* *p* < 0.05). (**b**) Western blot analysis of α7 nAChR protein expression in SCs after 48 h of 10 μM ICH3 treatment. SCs were pretreated with BK for 24 h before ICH3 treatment. β-Actin was used as the internal reference protein. The graph shows the densitometric analysis of the α7 nAChR immunopositive bands revealed by Western blots normalized against the bands of β-Actin used as the internal reference protein. Data are the average ± SEM of three independent experiments. *T*-test’s student was used to compare the samples (** *p* < 0.01).

We have previously shown that under basal conditions SCs do not express the *α*7 nAChR, whereas this receptor subtype is upregulated when an inflammatory stimulus is provided [11]. As shown in Figure 1, receptor activation with ICH3 treatment causes a significant increase in *α*7 nAChR expression both at transcriptional (a) and protein levels (b). This could indicate an autoregulation of receptor synthesis, since the *α*7 nAChR is already upregulated in inflammatory microenvironment. Through Western blot analysis, we showed that SCs also express other nAChR subunits, such as β4, *α*3 and β3, indicating the coexistence of *α*7 nAChR with other nicotinic receptor subtypes. Their protein levels, however, were not increased by either BK or ICH3, but, instead, both treatments induced a significant reduction In the levels in particular of the *α*3 and β3 nAChR subunits (Figure S1).

### 3.2. [Ca^2+^]_i_ Measurements in Schwann Cells

**Figure 2:**
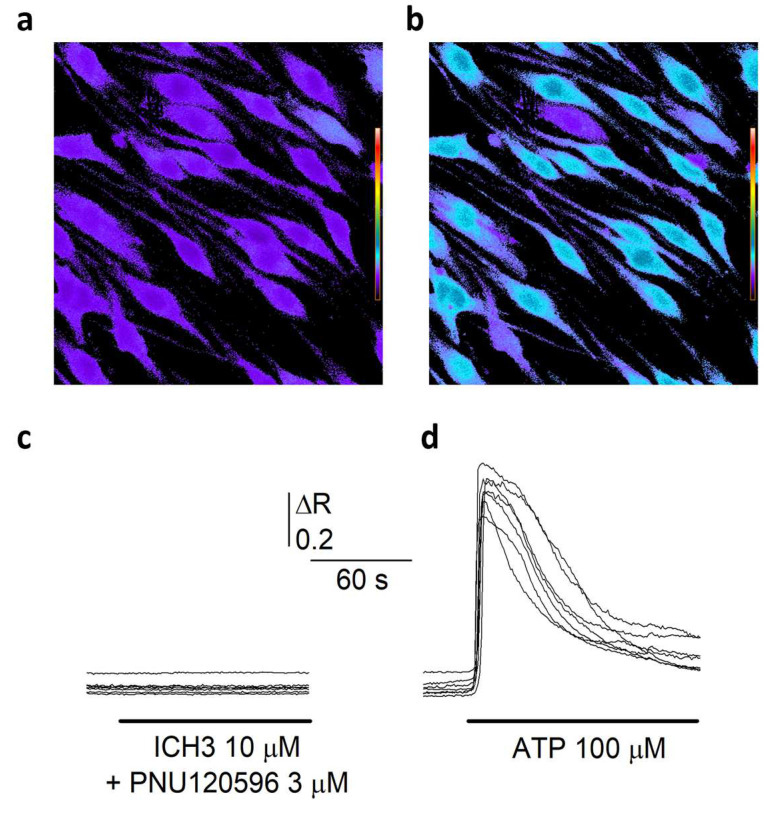
α7 nAChRs do not mediate Ca^2+^ entry in SCs. (**a**) Typical fluorescence microscopy optical field showing Fura-2 loaded in SCs at rest. [Ca^2+^]i is represented by pseudo colors, according to the calibration scale on the left. (**b**). The same cells reported in (**a**). after ATP application (100 μM, no wash). It is possible to note the strong [Ca^2+^]i elevation in most cells. (**c**) Time course of the [Ca^2+^]i recorded in 7 cells during co-application of the α7 selective partial agonist ICH3 (10 μM) and the α7 PAM PNU-120596 (3 μM). It is to note the absence of Ca^2+^ mobilization. (**d**) Time course of the [Ca^2+^]i recorded in 7 cells during the application of ATP 100 μM. Please note the large and transient [Ca^2+^]i elevations.

Since the homomeric α7 channel is characterized by a high Ca^2+^ permeability among the nAChR subtypes [21], its activation might mediate a direct Ca^2+^ entry in SCs. To verify this hypothesis, we measured, by time-resolved digital fluorescence microscopy, the changes in intracellular free Ca^2+^ concentration ([Ca^2+^]_i_) induced by the application of different nicotinic agonists in SCs loaded with the Ca^2+^ sensitive Fura-2 dye (Figure 2a). No response was observed upon ACh administration (1 mM), in the presence of 10 μM atropine to avoid muscarinic responses (48 cells, 3 experiments, not shown). As a positive control, 100 μM ATP application induced very clear Ca^2+^ transients in SCs (Figure 2b,d). Similarly to ACh, the α7 selective partial agonist ICH3 (10 μM) did not elicit any intracellular Ca^2+^ response. Furthermore, co-application of both ACh and ICH3 with the selective α7 positive allosteric modulator PNU-120596 (3 μM) [22] did not induce any Ca^2+^ transient (Figure 2a,c), indicating that α7 nAChRs as well as other nAChRs eventually expressed by SCs do not mediate any increase of [Ca^2+^]_I_ in these cells.

### 3.3. Analysis of the PI3K/AKT/mTORC1 Pathway

Although α7 ion channels behave as conventional ionotropic receptors, as reported above their stimulation with both ACh and α7 activator ligands may fail to increase [Ca^2+^]_i_ levels. These results suggest that a non-canonical signaling may be associated with nAChRs [23] and that the use of partial agonists can promote a metabotropic-like activity of the α7 nAChRs [24]. Therefore we investigated whether receptor activation could trigger and modulate a possible transduction pathway. A crucial pathway in the regulation of several processes in SCs is the PI3K/AKT/mTOR axis, where mTORC1 is the mainly involved complex [15,25,26]. In a previous study, we demonstrated that activation of the M2 mAChR negatively modulated this pathway, promoting a myelinating phenotype in SCs [9]. We wondered whether stimulation of the α7 nAChRs might also be involved in the regulation of this pathway. In each planned experiment, we previously increased the expression of the α7 nAChR subtype, which is very low at the basal level, by performing a 24 h pre-treatment with the inflammatory peptide BK [11]. Then BK was removed and SCs were treated with the partial agonist ICH3 for 48 h.

**Figure 3:**
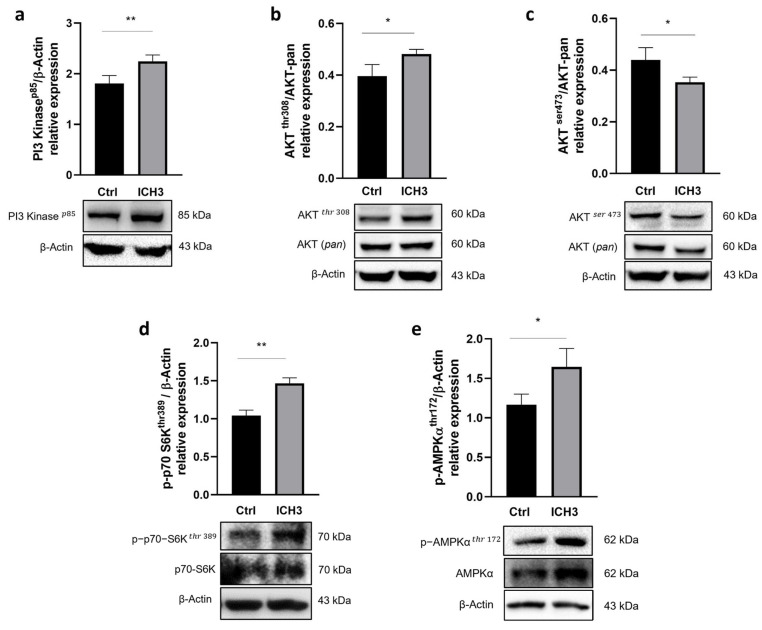
Western blot analysis of the PI3K/AKT/mTORC1 signaling pathway. SCs were treated with 10 μM ICH3 (**a**) PI3K expression. β-Actin was used as internal reference protein. The graph shows the densitometric analysis of the bands of Western blot analysis for PI3K normalized with the bands of the reference protein β-Actin. (**b**) Phospho-AKT (Thr308) expression. β-Actin was used as internal reference protein. The graph shows the densitometric analysis of the bands of Western blot analysis for Phospho-AKT normalized with the bands of the AKT (pan) protein. (**c**) Phospho-AKT (Ser473) expression. β-Actin was used as internal reference protein. The graph shows the densitometric analysis of the bands of Western blot analysis for Phospho-AKT normalized with the bands of the AKT (pan) protein. (**d**) Phospho-p70S6K (Thr 389) expression. β-Actin was used as internal reference protein. The graph shows the densitometric analysis of the bands of Western blot analysis for Phospho-p70S6K normalized with the bands of the non-phosphorylated p70S6K protein. (**e**) Phospho-AMPK*α* (Thr172) expression. β-Actin was used as internal reference protein. The graph shows the densitometric analysis of the bands of Western blot analysis for Phospho-AMPK*α* normalized with the bands of the non-phosphorylated AMPK*α* protein. All data are the average ± SEM of three independent experiments. Student’s *t*-test was used (* *p* < 0.05; ** *p* < 0.01).

Protein levels of PI3K p85 protein expression, evaluated by Western immunoblot were significantly increased upon 48 h treatment with 10 μM ICH3 (Figure 3a). Because the main downstream effector of PI3K is AKT, we explored whether its two phosphorylated forms, AKT^Thr308^ and AKT^Ser473^, could also be modulated. As shown in Figure 3b,c, we observed an increase of p-AKT^Thr308^ protein levels but a decrease in those of p-AKT^Ser473^. Since phosphorylation at AKT^Thr308^ alone is sufficient to activate its targets [27], we investigated whether mTORC1 was activated by evaluating the expression of its direct target, p-p70 S6K^Thr389^. Western blot analysis showed increased levels of phosphorylated form of this kinase (Figure 3d). As the phosphorylation of AKT on Ser473 is carried out by the mTORC2 complex [28], its significant reduction (Figure 3c) following α7 nAChR stimulation suggests a concomitant reduction in mTORC2 activity. Since the expression of the α7 nAChR increases following an inflammatory stimulus and the PI3K/AKT/mTORC1 pathway is activated under stimulation of this receptor subtype, we wondered whether the α7 nAChR could also promote a ‘repair’ phenotype in SCs. At this aim, we analyzed the expression of p-AMPK^Thr172^, a kinase which is considered a negative regulator of myelination, while its high levels promote c-Jun expression and cause down-regulation of myelin protein gene expression [29]. Our data show a significant enhancement of p-AMPK*α*^Thr172^ expression after 48 h exposure to 10 μM ICH3 (Figure 3e). To show that the upregulation of the PI3K/AKT/mTORC1 pathway and AMPK*α* protein expression had to be attributed to stimulation with ICH3, we also evaluated the protein levels of both p-p70 S6K^Thr389^, as a downstream factor of the pathway, and p-AMPK^Thr172^ also in cells stimulated with BK alone. Although this condition of stimulation caused upregulation of both kinases, their increased expression was significantly higher after ICH3 treatment (Figure S2).

### 3.4. C-Jun Expression in SCs after α7 nAChR Activation

**Figure 4:**
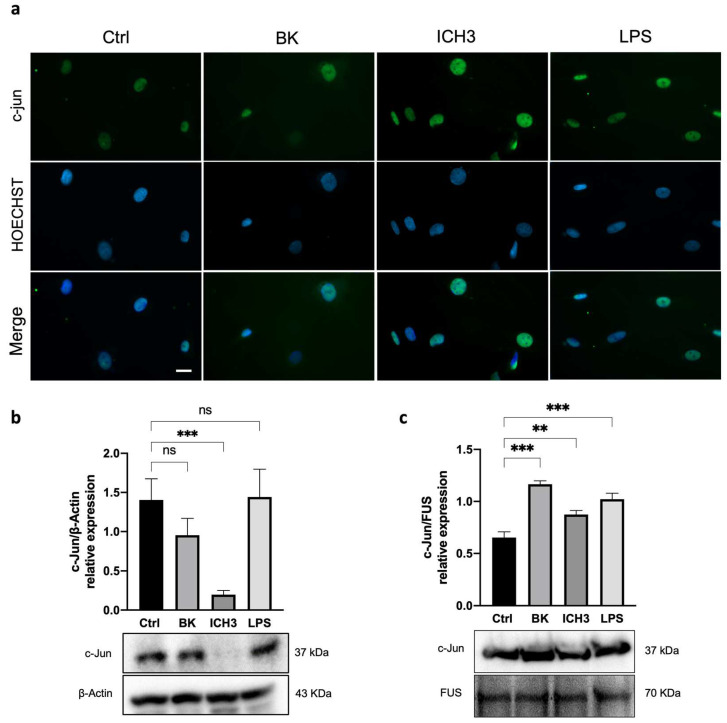
Analysis of c-Jun localization and expression in SCs after ICH3 treatment. (**a**) Immunocytochemistry analysis of SCs cells untreated or treated with 10 μM BK, 10 μM ICH3 (+10 μM BK), 100 ng/mL LPS for 3 h. Cells were immunostained with an anti-c-Jun (green) antibody and counterstained with Hoechst 33342 (blue). Scale bars: 10 μm. (**b**) Western blot analysis of protein expression of c-Jun in the cytoplasmic extracts of SCs after 24 h of treatment under the following experimental conditions: Ctrl (untreated cells), 10 μM BK, 10 μM ICH3 (+10 μM BK), 100 ng/mL LPS. β-Actin was used as cytoplasmic reference protein. Densitometric analysis showed in the graph was obtained from three independent experiments. One-way ANOVA test followed by the Tukey multiple comparison post-hoc test was used to statistically compare the different experimental conditions (*** *p* < 0.001; ns: not significant). (**c**) Western blot analysis of protein expression of c-Jun in the nuclear extracts of SCs after 24 h of treatment under the following experimental conditions: Ctrl (untreated cells), 10 μM BK, 10 μM ICH3 (+10 μM BK), 100 ng/mL LPS. FUS was used as nuclear reference protein. Densitometric analysis showed in the graph was obtained from three independent experiments. One-way ANOVA test followed by the Tukey multiple comparison post-hoc test was used to statistically compare the different experimental conditions (** *p* < 0.01; *** *p* < 0.001).

Among the various transcription factors modulating SC maturation and myelination, c-Jun has been shown to negatively regulate myelination, as it is activated and upregulated after axonal damage [30,31,32]. Since the increased expression of pAMPKα may be correlated with the increased expression of c-Jun, we evaluated the expression of this transcription factor in SCs after treatment with 10 µM ICH3. To promote the inflammatory microenvironment, the BK inflammatory signal was again used before treatment with the α7 nAChR agonist. We also utilized LPS as a positive control of an additional inflammatory stimulus and subsequent activation of c-Jun [33]. As shown in Figure 4, immunocytochemistry analysis showed the nuclear localization of c-Jun in all experimental conditions (Ctrl, 10 μM BK, 10 μM ICH3, and 100 ng/mL LPS). However, after 3 h of ICH3 treatment, the intensity of nuclear immunofluorescence was stronger than in the other samples (Figure 4a). To quantify the c-Jun protein, we performed a Western blot analysis by comparing its levels of expression in cytoplasmic and nuclear extracts obtained from SCs treated with ICH3 or LPS for 24 h (Figure 4b,c). Protein analysis of the cytoplasmic extracts showed the presence of the c-Jun protein in all experimental conditions except for the ICH3-treated samples (Figure 4b). In contrast, in the nuclear protein extract, c-Jun was observed in all experimental conditions (Figure 4c). However, compared with the untreated control, the c-Jun protein level in the nuclei significantly increased under the inflammatory stimulation, with both BK and LPS, as well as following α7 nAChR activation. The preferential localization of c-Jun in the nucleus is confirmed by immunofluorescence analysis and its absence in the cytoplasm extract after ICH3-mediated *α*7 nAChR activation indicate that the large part of protein translocates into the nucleus where it exerts its functions as a transcription factor.

### 3.5. Analysis of SC Migration after α7 nAChR Activation

**Figure 5:**
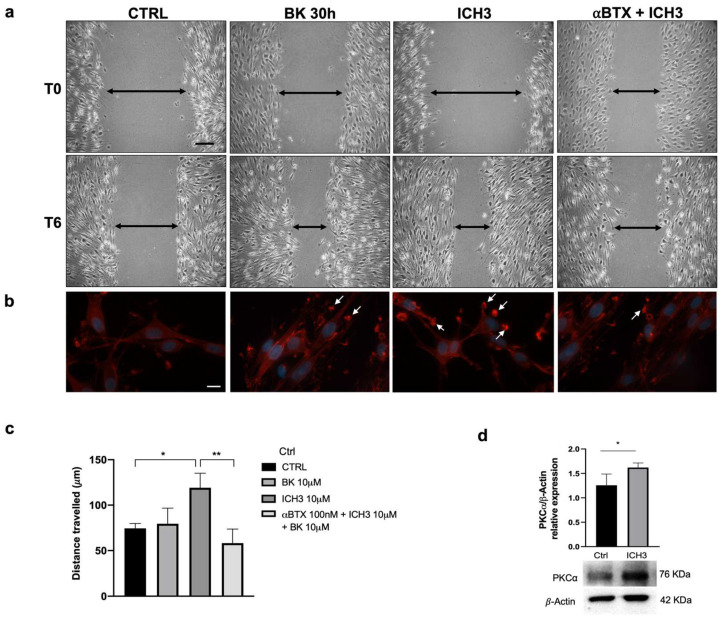
Cell migration after ICH3 treatment in SCs, assessed by a wound healing assay. (**a**) The images were obtained at time 0 (T0) corresponding to the time at which the scratch was performed, and after 6 h (T6), to analyze the scratch width under the following conditions: untreated cells (Ctrl), 10 µM BK, 10 µM ICH3 (after pretreatment with 10 µM BK), 100 nM *α*BTX + 10 µM ICH3 (after 24 h pretreatment with 10 µM BK), in SCs. The black arrows indicate the distance between two fronts. Scale bar: 100 µm. (**b**) Invadopodia-like formations (indicated by the white arrows) evaluated by actin staining with Alexa Fluor^TM^ 594 phalloidin in SCs maintained in the following experimental conditions: untreated cells (Ctrl), 10 µM BK, 10 µM ICH3 (after pretreatment with BK 10 µM), 100 nM *α*BTX + 10 µM ICH3 (after 24 h pretreatment with 10 µM BK). Hoechst 33342 was used for nuclei staining. Scale bar: 10 µm. (**c**) The graphs show the distance traveled, measured as the difference between the gap width at time 0 and after 6 h of treatment (T0–T6) for each experimental condition in SCs. The data are the average ± SEM of three independent experiments. One-way ANOVA test followed by the Tukey multiple comparison post-hoc test was used to statistically compare the different experimental conditions (* *p* < 0.05; ** *p* < 0.01). (**d**) Representative Western blot for the PKC*α* protein expression in SCs in the control condition and upon 48 h of treatment with 10 μM ICH3. β-Actin was used as internal reference protein. Densitometric analysis showed in the graph was obtained from three independent experiments. Student’s *t*-test was used to statistically compare the different experimental conditions (* *p* < 0.05).

To assess whether α7 nAChR activation affects the migratory capacity of SCs, we conducted a wound healing assay. Cells were pre-treated with BK for 24 h, then we provided ICH3 for additional 48 h. At the end of treatment, mechanical scratching was performed, and its amplitude was measured (T0). After additional 6 h (T6), the gap width was measured in cell cultures maintained under the different experimental conditions: Ctrl, 10 μM BK, 10 μM BK + 10 μM ICH3, 10 μM BK + 10 μM ICH3 + 100 nM αBTX (Figure 5a), where αBTX is an inhibitor of α7 nAChRs that acts as a competitive antagonist [34]. All measurements were compared with those taken immediately after scratching (T0). The graph in Figure 5c reports the distance travelled by the SCs under different treatment conditions, calculated as the difference between the initial gap width and that after 6 h from the scratching (T0–T6). Under all experimental conditions, we observed an increase in the migratory capacity of SCs; however, the distance traveled was significantly greater only after activation of α7 nAChR by ICH3. To prove that the altered migratory capability of SCs depends on the activation of α7 nAChR, we also treated the cells in the presence of the antagonist αBTX. Since the migration of SCs treated with αBTX + ICH3 significantly decreased compared with that of cells treated with ICH3 alone, we may conclude that the positive migratory effect is engendered by the activation of α7 nAChRs.

Since cell migration gives rise to morphological changes, among them membrane protrusions and variations in focal adhesions [35], we also evaluated the morphology of SCs under the different experimental conditions by assessing actin distribution by phalloidin staining (Figure 5b). After 48 h of 10 µM ICH3 treatment, several cell membrane extensions were observed, with aggregated actin in invadopodia-like structures. Spots of the same kind were observed at the periphery of the cells also in the other samples, whose dimensions were however reduced in comparison with those of the ICH3-treated cells (Figure 5b). Given changes in actin distribution as well as the increased migratory capacity of SCs following treatment with 10 μM ICH3, we evaluated the expression of the serine/threonine kinase PKC*α*, one of the pivotal kinases involved in these processes that is upregulated after nerve injury, especially during SC migration and proliferation [18,36].

As shown in Figure 5d, the Western blot analysis showed a meaningful overexpression of the PKCα protein after 10 μM ICH3 treatment, a result that confirms the ability of α7 nAChRs to activate this kinase and is in line with the increased migratory capacity of SCs.

## 4. Discussion

SCs are glial cells that actively participate in the regenerating processes. After the peripheral fiber lesions, these cells undergo a de-differentiation process which leads them to acquire a new phenotype, named “Repair Schwann Cells” [37]. Repair SCs exert strategic roles after nerve injury since they are involved in myelinophagy events, in regulating inflammatory processes, in neurotrophic factors production, and in driving regenerating axons through the Büngner bands formation [38]. Recently, we demonstrated that in SCs the expression of α7 nAChRs, which is faint in basal conditions, is significantly increased after peripheral axotomy or in inflammatory conditions [11]. In an in vitro model of the axotomized sciatic nerve, α7 nAChR selective activation by the partial agonist ICH3 caused a decreased release of the pro-inflammatory cytokine IL6 and promoted the MMP2 and MMP9 activities, suggesting a role of this receptor subtype in modulation of extracellular matrix and reestablishment of tissue homeostasis after peripheral damage [11,39]. For a more in-depth investigation of the role exerted by α7 nAChRs during peripheral nerve regeneration, we studied the signaling pathways downstream of their activation as well as the effects produced on SCs.

Our experimental model is represented by cultured SCs from sciatic nerves that were pre-treated with the inflammatory peptide bradykinin (BK) for 24 h, to promote α7 nAChR expression [11]. In this work, we showed that following stimulation with ICH3 also markedly increased the expression of nAChR α7 in SCs, suggesting an autocrine regulation mechanism able to ensure receptor availability in the “Repair SCs” during peripheral regeneration. Schwann cells also express other nicotinic receptor subunits such as α3, 𝛽3, 𝛽4 nAChR. By Western blot analysis we demonstrated their presence, however their expression results were downregulated both by BK and ICH3 stimulation (see Supplementary Figure S1), in contrast to what was observed for the α7 nAChR subtype (Figure 1) [11]. Their downregulation may be due to the direct or indirect effects of ICH3 or as a compensatory effect of the α7 subtype increase. However, the low expression and their downregulation after our treatments would exclude their primary involvement in the ICH3-mediated effects. Although it is not possible to exclude the presence of other nicotinic receptor subtypes, a previous report demonstrated that α9 and α10 nicotinic subtypes are not expressed in SCs.

Although α7 nAChRs behave predominantly as ionotropic receptors, in our experiments we did not observe any calcium mobilization when SCs underwent a combined treatment with two selective α7 nAChR activator ligands, i.e., the orthosteric agonist ICH3 and the selective positive allosteric modulator PNU-120596. Similar effects were also observed after ACh treatment in combination with muscarinic antagonist atropine, confirming that neither α7 nAChR or other nicotinic receptor subtypes are able to increase intracellular calcium levels in SCs. Conversely, upon 100 μM ATP treatment, SCs displayed a significant increase of Ca^2+^ transients. Thus, the lack of calcium mobilization by nicotinic receptor activation in general and α7 nAChRs in particular, suggests that they might trigger metabotropic signaling pathways, as observed in other non-neuronal cells [40,41].

**Figure 6:**
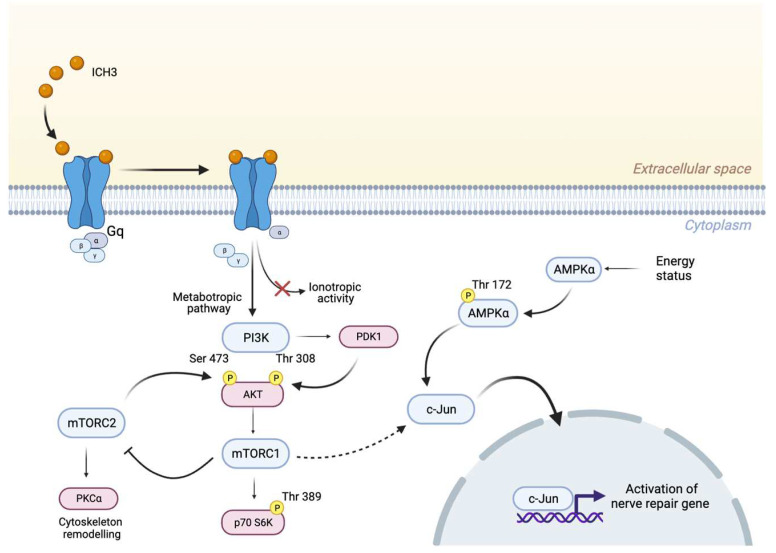
Schematic representation showing the metabotropic signaling activated downstream α7 nAChRs by the partial agonist ICH3. The activation of mTORC1 and AMPKα positively regulates c-Jun transcription factor, promoting the regenerative processes in Schwann cells.

In the search for the signaling transduction pathways involving the α7 nAChRs activation, we considered previous data obtained with mAChR ligands, and by a first Western blot analysis, we observed a significant increase of PI3K protein levels, followed by enhanced levels of pAKT^Thr308^ and a reduced expression of pAKT^Ser473^. These results are in agreement with literature data reporting that increased levels of pAKT^Thr308^ have been found after axonal damage [16]. In addition, since activation of the PI3K/AKT pathway may promote mTORC1 complex activation, we analyzed this signaling pathway downstream of α7 receptor stimulation. Interestingly, the modulation of p-p70 S6K^Thr389^, the main effector downstream TORC1 pathway, such as AMPK expression, was significantly increased by BK but their levels of expression were significantly higher following ICH3 treatment (Figure 3d,e and Supplementary Figure S2), confirming a prominent role of the α7 receptor in the modulation of this signal transduction pathway. Since adult SCs are characterized by low levels of mTORC1, the enhanced expression of the latter appears to be necessary for the correct response to axon damage. In fact, on the one hand, mTORC1 is involved in an autophagy process defined as myelinophagy, while on the other hand it promotes the increase of factors, such as the transcription factor c-Jun, favoring the regenerative properties of SCs [16]. In line with these observations, we found that α7 receptor activation promotes an increased nuclear expression of c-Jun, as shown by immunocytochemistry and Western blot analysis on nuclear SC extracts. The increased nuclear expression of c-Jun following α7 nicotinic agonist treatment was comparable with that observed in the presence of BK and LPS, two main inducers of inflammatory processes; however, after α7 nAChR stimulation, we observed a complete translocation of the c-Jun protein into the nuclei. At the same time, we found that α7 receptor activation produces significantly increased levels of p-AMPk^Thr172^, one of the main negative modulators of myelin formation. Altogether, these data suggest that α7 nAChRs, as proposed for other non-neuronal cells (i.e., immune cells and microglia), activate metabotropic signaling probably via βγ subunits of Gq [42,43]. This signaling pathway may contribute to promoting the “Repair Schwann Cells” phenotype, improving the regenerative properties (i.e., c-Jun nuclear translocation) of SCs and counteracting the stabilization of myelin sheath (Figure 6).

In addition, regenerating SCs could rescue their proliferative ability and increase their migration directed at forming the Büngner bands, both these properties being necessary to stimulate and drive axon regeneration [44]. In our experimental conditions, however, the α7 nAChR activation did not promote cell proliferation [45]. This result is apparently in contrast with the acquisition of the repair phenotype by SCs. It is worth mentioning, however, that cultured SCs are already proliferating and express basal levels of c-Jun, as is also evident by our data. It is therefore plausible that in our experimental conditions, activation of α7 nAChRs, although sufficient to promote the translocation of c-Jun in the SCs nuclei, could fail to induce an additional increase of cell proliferation.

On the other hand, our experimental protocol allowed us to estimate the increase in cell migration after α7 nAChRs stimulation, as shown in the wound healing assay. The co-treatment with mitomycin, which arrests cell proliferation, confirmed that the observed effects are dependent on cell migration [46]. Conversely, α-bungarotoxin, the preferential α7 nAChR antagonist, counteracted the effect of the nicotinic agonist ICH3, thus demonstrating that the enhanced cell migration is directly related to the α7 nAChR subtype activation. The phalloidin staining test, evidencing the increased production of lamellipodia, and the enhanced expression levels of PKCα, a typical positive modulator of the cytoskeleton remodeling during cell proliferation and migration [18,36], further reinforced the contribution of α7 nAChR activation in the modulation of migrating properties of SCs.

## 5. Conclusions

The results herein discussed, in accordance with our previous data obtained in cultured sciatic nerve, confirm a relevant role of α7 nAChRs in SCs. The peculiar expression of this receptor subtype in SCs only after peripheral nerve injury clearly suggests that acetylcholine, which is probably released by SCs themselves when isolated from the axons [47], may contribute, via preferential α7 nAChR stimulation, to modulate the regenerative properties of SCs by potentiating the “Repair Schwann Cells” phenotype, addressing axon regeneration, and regulating the inflammatory microenvironments. These data may have a clinical relevance in the treatment of severe peripheral nerve injuries, which should require, in addition to surgical therapy, a pharmacological treatment aimed to favor the fast recovery of peripheral sensory and motor functions.

## Data Availability

Not applicable.

## Supplementary Materials

The following supporting information can be downloaded at: https://www.mdpi.com/article/10.3390/cells12111494/s1, Figure S1: Expression levels of β4, α3, β3 nAChR subunits in SCs; Figure S2: Phospho-p70S6K^Thr 389^ and Phospho-AMPKα^Thr 172^ expression in SCs after BK and ICH3 treatment.
