# Supplementary material for: α7 Nicotinic Acetylcholine Receptors May Improve Schwann Cell Regenerating Potential via Metabotropic Signaling Pathways
Source: Cells. 2023 May 28;12(11):1494. doi: 10.3390/cells12111494 (PMC10253098; doi:10.3390/cells12111494)
Supplement: Supplementary file 1 [file cells-12-01494-s001.zip › cells-2235345-supplementary.pdf]

Supplementary materials

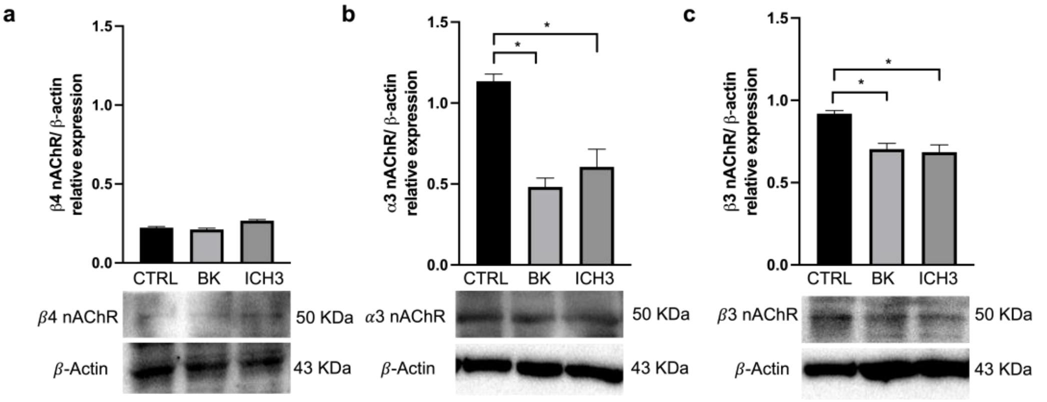

Figure S1. Expression levels of β4, α3, β3 nAChR subunits in SCs. Western blot analysis of **(a)** β4 nAChR, **(b)** α3 nAChR, **(c)** ~~β3~~ β3 nAChR protein expression in SCs after 24 h of treatment with 10 μM BK or 48 h of treatment with 10 μM ICH3 after 24 h of pretreatment with BK. SCs were pretreated with BK for 24 h before ICH3 treatment. β-Actin was used as internal reference protein. The graph shows the densitometric analysis of the bands of western blot analysis for α7 nAChR normalized with the bands of the β-Actin used as reference protein. The data are the average ± SEM of three independent experiments. T-test's student was used (*$p < 0.05$).

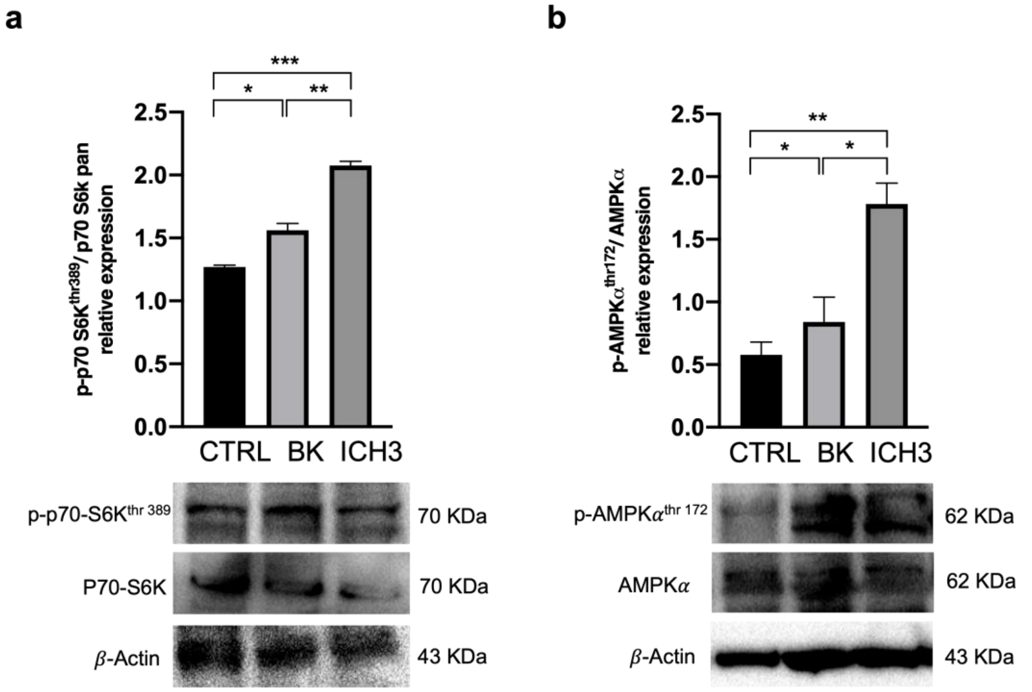

Figure S2. SCs were treated with 10 μM BK alone and with 10 μM ICH3 after BK pretreatment **(a)** Phospho-p70S6K$^{Thr\ 389}$ expression. β-Actin was used as internal reference protein. The graph shows the densitometric analysis of the bands of western blot analysis for Phospho-p70S6K normalized with the bands of the non-phosphorylated p70S6K protein. **(b)** Phospho-AMPKα$^{Thr\ 172}$ expression. β-Actin was used as internal reference protein. The graph shows the densitometric analysis of the bands of western blot analysis for Phospho-AMPKα normalized with the bands of the non-phosphorylated AMPKα protein. All data are the average ± SEM of three independent experiments. Student's t-test was used (*$p < 0.05$; **$p < 0.01$; ***$p < 0.001$).
